# Allied health assistants' perspectives of their role in healthcare settings: A qualitative study

**DOI:** 10.1111/hsc.13874

**Published:** 2022-06-10

**Authors:** Olivia A. King, Jo‐Anne Pinson, Amy Dennett, Cylie Williams, Annette Davis, David A. Snowdon

**Affiliations:** ^1^ Barwon Health Geelong Vic Australia; ^2^ Monash Centre for Scholarship in Health Education Monash University Clayton Vic Australia; ^3^ Monash Health, Department of Medical Imaging Clayton Vic Australia; ^4^ Peninsula Health, Department of Medical Imaging Frankston Vic Australia; ^5^ Department of Medical Imaging and Radiation Sciences Monash University Clayton Vic Australia; ^6^ Allied Health Clinical Research Office Eastern Health Box Hill Vic Australia; ^7^ School of Allied Health Human Services and Sport La Trobe University Bundoora Vic Australia; ^8^ School of Primary and Allied Health Care Monash University Frankston Vic Australia; ^9^ Academic Unit, Peninsula Health Frankston Vic Australia; ^10^ Allied Health Workforce Innovation Strategy Education Research (WISER) Unit Monash Health Clayton Vic Australia; ^11^ Peninsula Clinical School, Central Clinical School Monash University Frankston Vic Australia; ^12^ National Centre for Healthy Ageing Frankston Vic Australia

**Keywords:** allied health assistants, qualitative research, health workforce, organisation and administration.

## Abstract

Allied health assistants (AHAs) are important members of the health workforce and key to meeting population health needs. Previous studies exploring the role and utility of AHAs from multiple stakeholder perspectives suggest AHAs remain poorly utilised in many healthcare settings. This qualitative study explores the experiences and perspectives of AHAs working in healthcare settings to determine the contextual factors influencing their role, and mechanisms to maximise their utility. We conducted semi‐structured interviews using purposive sampling with 21 AHAs, from one regional and three metropolitan health services in Australia, between February and July 2021. We used a team‐based framework approach to analyse the data. Four major themes were identified: 1) AHAs' interpersonal relationships, 2), clarity and recognition of AHA roles and role boundaries, 3) AHAs accessing education and professional development, and 4) the professional identity of the AHA workforce. Underpinning each of these themes were relationships between AHAs and other healthcare professionals, their patients, health services, and the wider AHA workforce. This study may inform initiatives to optimise the utility of AHAs and increase their role in, and impact on, patient care. Such initiatives include the development and implementation of guidelines and competencies to enhance the clarity of AHAs' scope of practice, the establishment of standardised educational pathways for AHAs, and increased engagement with the AHA workforce to make decisions about their scope of practice. These initiatives may precede strategies to advance the AHA career structure.

**What is known about this topic?**

1. Allied health assistants (AHAs) are essential members of contemporary healthcare teams and services, as they strive to manage increasing demand for their services
2. AHAs' roles and functions within healthcare teams are limited by inconsistent or inappropriate task delegation
3. Allied health professionals are reluctant to delegate tasks to AHAs for numerous reasons related to their knowledge, skills, and experience and their relationships with AHAs

**What does this paper add?**

1. AHAs' roles are shaped by their interpersonal relationships with delegating AHPs, the broader healthcare team, other AHAs, and the patients they work with
2. AHAs' roles are health service factors including their access to education and professional development, the clarity and recognition of their roles, and role boundaries
3. AHAs perceive a need to develop and progress the identity of their workforce to maximise their contribution to healthcare teams and patients

## 1 INTRODUCTION

The health assistant workforce is growing and becoming increasingly entrenched in care teams across numerous health and social care settings (Hooker et al., 2019; Mickan et al., 2018; Moran et al., 2011; Moran et al., 2015). In Australia, allied health assistants (AHAs) are critical to both the local health service and broader policy‐level response to increasing and enduring health workforce shortages (New South Wales Ministry of Health, 2020; Somerville et al., 2015, 2018). AHAs work with allied health professionals (AHPs) under their delegation and supervision to provide a range of allied health services (Huglin et al., 2021; Snowdon et al., 2020). The allied health professions comprise a range of autonomous healthcare professions that can be categorised as therapy disciplines (e.g., dietetics, occupational therapy, physiotherapy, podiatry) and science disciplines (e.g., audiology, optometry, pharmacy, radiography) (Department of Health & Human Services, 2016). AHAs provide direct therapeutic services, coordination of group therapy, and administrative and other non‐clinical support services (Huglin et al., 2021; Moran et al., 2015).

AHAs play a fundamental role in healthcare delivery and yet remain underutilised in many health and social care settings. In part, this is due to inconsistent and inappropriate delegation, limiting AHAs' opportunities to practice to their full scope (Huglin et al., 2021; Nancarrow et al., 2013; Rushton et al., 2021; Somerville et al., 2018). There are numerous reasons underlying the inadequate delegation including: incompatible workforce structures (Somerville et al., 2018); immature relationships between delegating AHPs and AHAs; AHPs' lack of knowledge of confidence or trust in AHAs' abilities; AHPs' lack of confidence in their ability to undertake or supervise the clinical task (Brown et al., 2020; Nancarrow et al., 2013; Somerville et al., 2015); and a reluctance to relinquish part of the AHP role domain (Mickan et al., 2018; Nancarrow et al., 2013).

Optimal utilisation of the AHA workforce is critical to meeting health workforce challenges that relate to shifting demographics, modernised models of care that emphasise patient participation, and chronic workforce shortages (King et al., 2015; Nancarrow, 2015). Accordingly, there has been increased interest in developing evidence‐based strategies to maximise the utilisation of the AHA workforce. Previous studies have explored the AHAs’ role (Lizarondo et al., 2010; Stanhope & Pearce, 2013); AHPs' role in delegating to and supervising AHAs (Brown et al., 2020), and ways to optimise the use of AHAs' skills to support AHPs (Huglin et al., 2021; Moran et al., 2015; Somerville et al., 2015, 2018). These studies, however, are limited by either the non‐inclusion of AHAs as participants, or by the inclusion of AHAs in combination with other stakeholder groups without distinction of AHAs' unique perspectives and contributions. This may explain why the identified reasons for underutilisation of AHAs predominantly relate directly to the delegating AHP. A deeper understanding of AHAs' perspectives may identify new insights on how to better utilise the AHA workforce. Moreover, previous studies of AHAs' roles have been conducted across different contexts, including health and social care settings (Huglin et al., 2021; Moran et al., 2015). It is recognised that healthcare settings are unique and complex environments, characterised by professional hierarchies and social, political, and other organisational factors that influence practice change (Rogers et al., 2020). Therefore, the key factors that shape AHAs' roles in healthcare settings, according to the experiences and perspectives of AHAs themselves, are not yet clear. To develop comprehensive evidence‐informed strategies to optimise the skills, role, and utilisation of AHAs, it is essential that we address this evidence gap.

The aim of this study was to explore the experiences and perspectives of AHAs working in healthcare settings, and to address the following research questions:

1. What are AHAs' perspectives of the contextual factors that shape their roles in healthcare settings?
2. How do AHAs perceive they can be supported to maximise their utility in healthcare settings?

## 2 MATERIALS AND METHODS

### 2.1 Setting

This study was set in Victoria, Australia, where publicly funded health services operate independently of one another. AHAs from one regional and three large metropolitan health services were invited to participate in the study. Data were collected between February and July 2021.

### 2.2 Study design and participants

This qualitative study was underpinned by social constructionism which recognises knowledge is subjective; generated and shared through social interactions (Varpio et al., 2020). With multi‐site ethics approval from the lead health service (RES‐20‐0000118 L), AHAs working in four health services were recruited. AHAs were recruited either by email distributed via health service AHA lists, or in person at departmental meetings. AHAs interested in participating then contacted a member of the research team via email and provided written informed consent prior to their interview.

Individual semi‐structured interviews were conducted either in person or via telephone, by one of four authors (JP, AD, DS, or OK). We used a flexible interview guide to ensure consistency and relevancy of the data collected and enable free‐flowing discussion for rich data to be generated (King, 2021). The interview questions (Supplementary File 1) prompted participants to consider the team, health service, and other contextual factors that shape their role as an AHA. Participants were also asked to consider how they could be supported to maximise their role in their health service.

We analysed the data using a five‐stage framework approach which involved three authors becoming familiarised with the data and conducting a cursory analysis of three transcripts each (JP, DS, and OK). One author (OK) developed an initial coding framework, which was reviewed by the analysis team. One author coded all data (OK) and another author (DS) cross‐checked five transcripts. Data were charted to identify patterns which were then mapped and interpreted in the context of existing literature (Ritchie & Spencer, 1994).

Prior to commencing the data analysis, we conducted a reflexivity exercise that provided clarity with respect to our clinical, managerial, and research experience, expectations of the study, and our relevant theoretical perspectives. Our analysis team comprised of clinical AHPs who had worked with AHAs, clinician‐researchers, and one who had supported an emerging AHA‐researcher. The wider research team included senior allied health researchers and managers. We optimised the utility of our diverse and relevant experiences and perspectives throughout the analysis process. None of the interviews were conducted by a researcher who had a direct or managerial relationship with participants.

## 3 RESULTS

Twenty‐one individual interviews with AHAs were conducted. Interviews lasted between 19 and 51 minutes with approximately 13 hours of data collected in total. See Table 1 for participants' setting and delegating profession. Participants had between 6 months and 21 years of experience as an AHA. Six different delegating allied health professions were represented.

**TABLE 1:** Participants' setting and delegating profession

Participant no.	Setting	Delegating profession
1	Inpatient	Physiotherapy
2	Inpatient	Physiotherapy
3	Inpatient	Physiotherapy
4	Mixed	Medical imaging
5	Community	Physiotherapy
6	Community	Multidisciplinary (physiotherapy and occupational therapy)
7	Community	Multidisciplinary (physiotherapy, occupational therapy, and speech pathology)
8	Inpatient	Multidisciplinary (physiotherapy and occupational therapy)
9	Mixed	Medical imaging
10	Mixed	Medical imaging
11	Inpatient	Social work
12	Inpatient	Speech pathology
13	Inpatient	Dietetics
14	Inpatient	Multidisciplinary (physiotherapy, occupational therapy, and dietetics)
15	Mixed	Medical imaging
16	Mixed	Multidisciplinary (physiotherapy, occupational therapy, dietetics, and social work)
17	Inpatient	Multidisciplinary (physiotherapy, occupational therapy, dietetics, and social work)
18	Inpatient	Occupational therapy
19	Mixed	Multidisciplinary (physiotherapy, occupational therapy)
20	Inpatient	Physiotherapy
21	Inpatient	Multidisciplinary (physiotherapy and occupational therapy)

*Note*: Six were employed as junior or mid‐level AHAs and 11 were employed as advanced practice or senior‐level AHAs. Grading of Medical Imaging AHAs differs from therapy AHAs and are classified under various health service awards.

**FIGURE 1:**
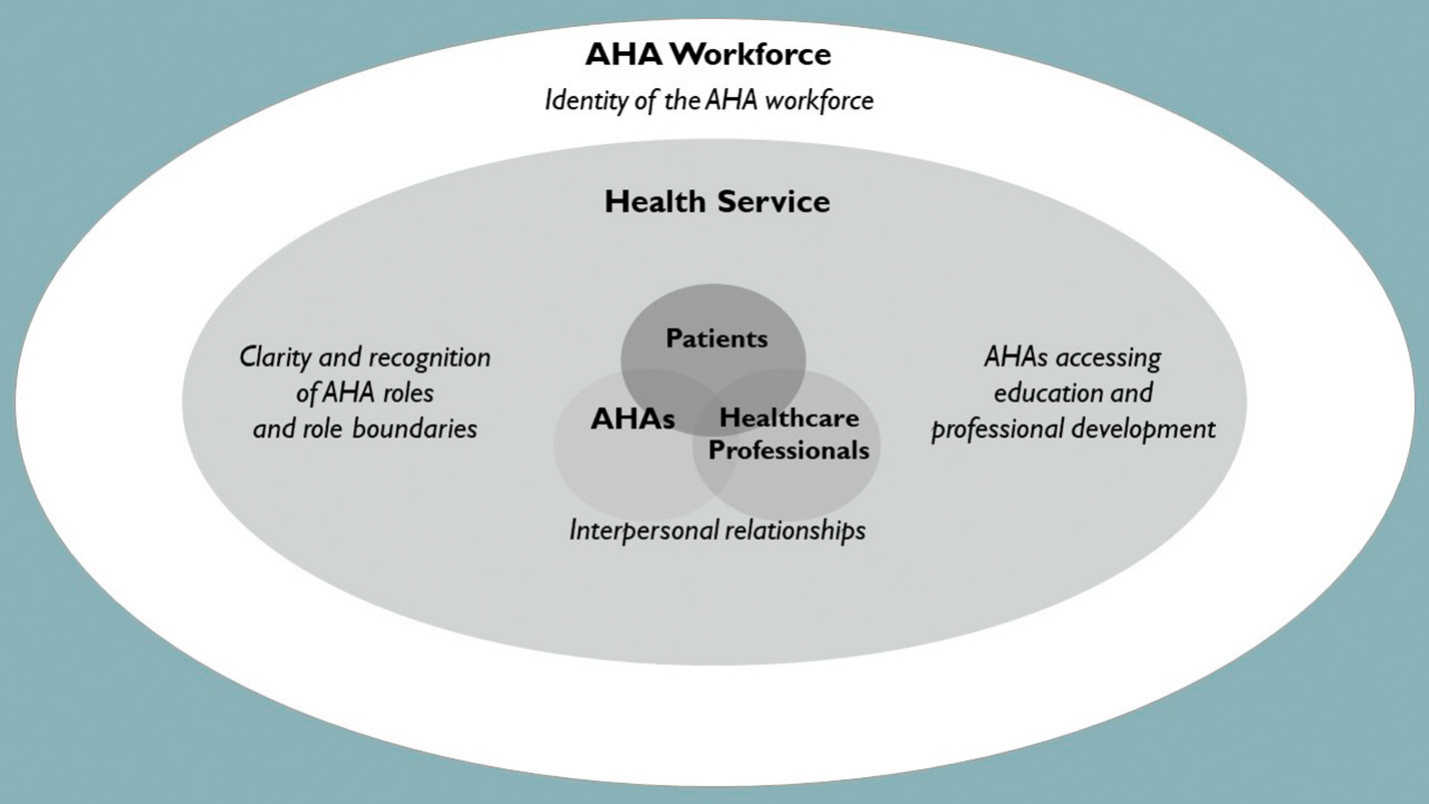
Key themes and underpinning contextual factors

Four major themes were identified: 1) AHAs' interpersonal relationships, 2), clarity and recognition of AHA roles and role boundaries, 3) AHAs accessing education and professional development, and 4) the professional identity of the AHA workforce. Each theme sits within the context of the AHA and their relationship with 1) healthcare professionals and patients, 2) the health service, and 3) the wider AHA workforce (Figure 1). These themes are described in detail below with illustrative participant quotes. Ellipses have been used where quotes have been abbreviated and square brackets denote additional text inserted to provide context.

### 3.1 AHAs' interpersonal relationships: “Developing trust”

AHAs foster multiple professional relationships through communication, rapport‐building, and a commitment to teamwork, with a view to providing patient‐centred and effective care. Participants referred to their relationships with various individuals within the healthcare context as key to shaping their role. These included relationships with delegating AHPs, other AHAs, members of the wider healthcare team (including referrers to services), and to the patients and their families. Trust, clear, consistent, and considered communication were critical to these relationships:

“… part of developing this [AHA] job is meeting these people, developing trust and fleshing out the role … There are some delegations I've had from the leads because I've got good rapport with them … I have to be in their face, and my profile has to be up so that they will think of me. It's exhausting.” (Participant 14, Inpatient Multidisciplinary AHA)

AHAs need to manage their profile and demonstrate their value as a member of the healthcare team through consistent communication and rapport‐building with potential referrers. Positive and regular interactions over time aided the development of trust with their delegating AHPs:

“I think most of the social work team appreciate that …. of course they can do what I do but it's that follow‐up and just them knowing, ‘good, it's done’, and [they] know it will be done to a good standard. That's something that develops over time within your team, that trust.” (Participant 11, Inpatient Social Work AHA)

Some participants, however, accepted that there is a subset of AHPs who will not delegate for different reasons including under‐developed relationships or incompatibility between the AHP and the AHA, the level of seniority of the AHP including confidence in their own clinical and delegation skills, and simply, AHPs' personal preference:

“… you don't always mesh with every single person and some of them like to do things their own way, so they'll take over sometimes.” (Participant 15, Mixed Settings, Medical Imaging AHA)

The need to develop and nurture relationships with other key individuals within the healthcare setting were described (e.g., with doctors and nurses) and communication appeared to be pivotal to these relationships, as the next participant describes:

“I spend a lot of time liaising with nursing staff, we have timetables in our patients' rooms, and I have timetables in nursing handover, to make sure that everyone's on the same page.” (Participant 21, Inpatient Multidisciplinary AHA)

Participants also described their relationships with other AHAs and the need for enhanced teamwork and support. Where some benefitted from having other AHAs in their team or setting, others felt professionally isolated:

“If you're working with someone … you can get a good role going, and that makes your day. For [AHA colleague] working on home‐based, she finds it quite isolating. Even though you're working with other professionals, it's not the same as working with someone actually in your same role.” (Participant 17, Inpatient Multidisciplinary AHA)

Participants valued relationships with their patients which was achieved through sound communication and rapport‐building: “I feel a patient will tell us more than sometimes what they'll tell you. … You build up that bond” (Participant 17, Inpatient Multidisciplinary AHA).

AHAs emphasised the importance of their direct engagement with patients through their intense and consistent relationships during their treatment or rehabilitation phase, ensuring they benefit from continued therapeutic support:

“I think my role there adds continuity of care from the physio that might see them once every day, or second day, third day, depending on their workload, and where they [patient] sit on the priority list. I see these patients every day from day zero. … until they're discharged, so they get that one person that's consistent.” (Participant 8, Inpatient Multidisciplinary AHA)

Numerous participants shared this sentiment. They viewed the therapeutic aspect of their role as more important than the administrative aspect and expressed a willingness to expand their role in providing patient care.

### 3.2 Clarity and recognition of AHA roles and role boundaries: “Knowing our skills and competencies”

Participants described varying levels of clarity, recognition, and understanding of their role and role boundaries by their delegating AHPs. Participants frequently emphasised their own recognition of their role boundaries and limits in terms of their scope of practice, at times, being reinforced over time by their delegating AHPs or team members:

“It's probably been drummed into me. Knowing my capabilities and knowing that I take the direction from the clinician that I'm working with. If I need to ask that clinician any questions or I have any concerns, I know not to just make up answers to clients, and to go back and check with the clinician.” (Participant 6, Community Multidisciplinary AHA)

Those with sufficient role clarity appeared to experience more structure around their role and with that comes greater autonomy. Autonomy and structure were also emphasised by appropriate delegation to and support for the AHA as required:

“We as AHAs do have a great deal of autonomy within our role within the overall wider team. We're not supervised on a day‐to‐day basis but the physiotherapists are on hand as a resource for any questions on medical status or treatment options. We carry our own caseload.” (Participant 3, Inpatient Physiotherapy AHA)

Some AHAs had a clearly defined role within the team, which was determined by the delegating AHP. Others described having less clarity and understanding of their role, particularly among the broader healthcare team.

“There's a bit of debate on whose role is whose. … Sometimes I feel I don't know what my role is or where I'm meant to be. … some of the nurses didn't even know what our roles were. … You get told one minute like, ‘No, you can't do that’. … and then the next minute you're told, ‘Well, why don't you just get on with it?’. … I find that very mixed.” (Participant 17, Inpatient Multidisciplinary AHA)

This ambiguity around the role of the AHA tended to be more common for multidisciplinary AHAs and contributed to the notion that AHAs are not utilised to their full capacity:

“The AHA role is so different and diverse across every different site, even in [Health Service]. I believe we could absolutely be utilised more in therapy. … I know the AHA role is mainly administration from my understanding. … which I think is a real waste of their skills. … It's just about knowing our skills and competencies.” (Participant 5, Community Physiotherapy AHA)

When the role of the AHA is unclear at the health service level, their utility across the various allied health disciplines is compromised. AHAs described having to actively demonstrate the nature of their roles, skills, and competencies to their immediate and broader teams to enhance the clarity and recognition of their role, and ultimately, promote appropriate delegation:

“we have to communicate [competency and skillset]. … my team knows. … but, other areas I don't know whether they do. I feel it's more up to the AHAs to give that information and let the AHPs know. It'd be really good to somehow have that communicated through the team” (Participant 5, Community Physiotherapy AHA)

The potential for competency attainment and assessment to improve the clarity of the AHA role was suggested by several participants. In turn, this may negate the need for AHAs to verbally promote their role and dedicate time and energy to nurturing trusting relationships with delegating AHPs. This appeared particularly relevant in the context of junior or rotating AHPs:

“If we actually do ensure that we tick off the competencies, when new Grade 1s rotate in and everything like that, then it's some kind of assurance. We can say, ‘Well, this AHA has competencies’. … and then that gives them more confidence [to refer the task].” (Participant 1, Inpatient Physiotherapy AHA)

Several participants described their role as critical in maintaining the workflow. In this respect, the value of the AHA was often highlighted when they were absent from the workplace:

“When we're not around, the physios really feel the difference and they've voiced that. Classic example, over Christmas when people take leave and when we return they say to us, "Oh, there was a day that I had to do my own [equipment] hires.” (Participant 2, Inpatient Physiotherapy AHA)

There were numerous references to the many “unseen things” AHAs do, or the many “incidentals” they engage in throughout their day. This unseen work that is essential to workflow makes the AHA role somewhat ambiguous and highlights the unquantifiable value of AHAs:

“If they replace me nothing gets done because the replacements don't really know my day‐to‐day activities. Even if I write it down of what I do, it changes day by day, because obviously it's not set. It can change within like half an hour, or it can change within five minutes.” (Participant 10, Mixed Settings Medical Imaging AHA)

AHAs also described ambiguity around their role in providing education to AHP students (i.e. providing orientation to the health setting) and colleagues, including AHPs and other healthcare team members. AHAs are often seen as resourceful people who have broad knowledge of the processes and changes happening within health services; for example, as this participant explains during COVID‐19:

“There's been lots of changes lately, and that puts a lot of pressure on AHAs, because they supposedly know everything. They can get bombarded with questions coming from everywhere, from new clinicians. … some questions, you feel [are] out of your scope, and you shouldn't be answering. … at the same time, you feel sorry for them, they have nowhere else to get the answers.” (Participant 6, Community Multidisciplinary AHA)

AHAs recognise the limitations imposed by their role boundaries but find it difficult to assert this to team members who need their assistance.

### 3.3 AHAs accessing education and professional development: “The best AHA I can be”

Participants made numerous references to their own education, training, and learning in the workplace. Owing to the changes in AHAs' systems of education, credentialing, and criteria for grading, there were inconsistencies in the formal education of the participants. Nonetheless, AHAs' own learning was primarily undertaken “on‐the‐job”:

“Throughout the years I've learned what's important and what's less important. … from the supervisors and the deputies training me on what to look for. Again, it's just acquired work learning throughout the years.” (Participant 10, Mixed Settings Medical Imaging AHA)

AHAs also described workplace learning through working with clinicians and highlighted opportunities to learn from proactive and inclusive AHPs, who are willing to share with and invest in their AHA team members. This was described as both incidental learning and intentional efforts by AHPs to train AHAs:

“The speech pathologists training me and taking that very seriously and being very proactive including me in any kind of learning opportunity.” (Participant 12, Inpatient Social Work AHA)

This quote highlights AHAs' opportunities to learn from proactive and inclusive AHPs, who are willing to share with and invest in their AHA team members. Other AHAs spoke about their opportunities to learn being shaped by the health service and the learning culture within:

“At [Site], OTs (occupational therapists) are fully staffed always and they have a great team and they have the opportunity to do some development sessions. … But, at [Site], for example, the OTs are very understaffed and maybe the leader or the senior is not very good. So that AHA is missing out on that professional development.” (Participant 20, Inpatient Physiotherapy AHA)

AHAs also described some frustration about their limited opportunities and choice when it comes to professional development. They explained that professional development opportunities are shaped by the organisational environment and are not always amenable to their identified learning needs or interests:

“There's a little bit of in‐house training, but I wouldn't say there's a lot of training. .. You do your course, you're plonked in and that's it.. . I've been trying to find stuff [professional development opportunities] I'd love to do, but you get knocked back.. . every day you're doing the same thing. It'd be nice to bring different.” (Participant 17, Inpatient Multidisciplinary AHA)

Participants frequently described a desire to learn and do more within their roles; either to provide more direct patient care, utilise the skills they already have, or to learn and master new skills to expand their role boundaries and scope of practice. This next participant, a medical imaging assistant, neatly captures their desire to learn and do more, the benefits, and the barriers to doing so:

“For future training I'd love to be able to do cannulation. That would save so much more time to do the cannulas. It would free up the nurses and time. It would be so much quicker to get through the scans. I've mentioned that to management but obviously there's a lot of red tape for that sort of thing.” (Participant 4, Mixed Setting Medical Imaging AHA)

Participants also highlighted the need for passionate AHAs to have access to opportunities to grow, develop, and remain engaged in their role:

“… in physio, they have split streams – neuro, ortho, women's health, paediatrics, or movement disorders. If we have that option to [take] those courses, then you could sign up for a weekend course and learn about that or wanted to move into a job. … I love my job – I would love to be able to be the best AHA I can be, and still learn, and still develop, and I think that when you get to a certain point, it kind of stops. I just love to learn.” (Participant 21, Inpatient Multidisciplinary AHA)

AHAs predominantly learn on the job and seek opportunities to expand their skillsets through informal workplace learning, training from AHPs, and formal professional development, so that they can maximise their contributions to their healthcare teams.

### 3.4 The identity of the AHA workforce: “It's developing”

Participants frequently expressed a love for their job, particularly the patient care aspects, employing terms such as “feeling privileged”, “enjoying”, or finding “joy” or “satisfaction” in their job. Yet they also articulated frustration with a lack of professional identity and perceptions of inferiority when compared to AHPs:

“Another thing I'd like is for the word ‘allied health assistant’ be removed and it would be ‘allied health colleague’. … I don't introduce myself as an AHA, I say I'm a physio colleague.

I'll explain it to [patients] because they sometimes think they're getting lesser treatment, they've been handed down from the physio to the assistant so they're going to get lesser treatment.” (Participant 3, Inpatient Physiotherapy AHA)

These feelings of frustration extended to how their roles and scope of practice are predominantly determined by AHPs. Numerous participants described the need for AHAs to have their own voice to shape their future careers and workforce:

“I think that when they talk about AHAs' jobs and our scope and what we do, traditionally, AHAs have actually not been very included. I think a lot of decisions about our job are made by clinicians, and not us. I'd like to see that stop.” (Participant 21, Inpatient Multidisciplinary AHA)

Strategies to enhance the AHA voice such as presenting at conferences on AHA matters, establishing an AHA lead representing organisation, and promoting unification and consistency among AHAs working across different health services were also described:

“I have gained a sense from talking with other AHAs at conferences that there is a difference of what one AHA does at one site compared to another. This seems to vary from health provider to health provider. AHAs are not registered as physiotherapy are, nor is there a regulatory body that governs this.” (Participant 3, Inpatient Physiotherapy AHA)

Participants were, however, optimistic that the AHA workforce was steadily progressing, with clearer career structures and opportunities for career progression. They described their perception of the evolution of the AHA role:

“It's developing and I'm hoping to see down the track, although I'll probably see it from afar, how it develops. … there's some hurdles for it to overcome for it to take the next step to where it goes for – whether they bring in a grade four role. … they've been talking about it for many years, or a Diploma of Allied Health Assistant, so that next structure.” (Participant 3, Inpatient Physiotherapy AHA)

There was a sense of frustration among AHAs who feel they have more to contribute to the teams and patients they work with, and that there has been a tradition of excluding AHAs in discussions about their current and future capacity. Participants have highlighted the need for AHAs to come together as an occupational group to enhance and progress their workforce.

## 4 DISCUSSION

To our knowledge, this is the first qualitative study that explores the contextual factors that shape AHAs' roles in healthcare settings, exclusively from the perspective of AHAs. AHAs perceive that their roles are influenced by individual (i.e. interpersonal relationships), health service (i.e. clarity on role boundaries and access to professional development), and broader workforce (i.e. identity of the AHA workforce) factors.

It is well established that the AHAs’ role is largely influenced by their relationships with AHPs (Huglin et al., 2021; Mickan et al., 2018; Somerville et al., 2015). Our findings extend this knowledge by highlighting that the AHAs’ role is also influenced by their relationships with other healthcare professions (e.g., nursing and medicine). Establishing these relationships, however, may be difficult for AHAs working within entrenched hierarchical social systems where status is in part determined by healthcare workers' knowledge and expertise (King et al., 2018). Within this hierarchy, healthcare workers with sub‐ordinate roles, such as AHAs, require high‐level communication skills to manage the expectations of those higher in the hierarchy (Apker et al., 2005). AHAs in our study provided examples of communication strategies, such as promoting their capabilities to healthcare professionals, demonstrating they have the capacity to effectively build and manage these relationships. Health services also have a role to play in promoting workplace environments that “flatten” the hierarchy and facilitate teamwork between healthcare workers (Braithwaite et al., 2016; Huglin et al., 2021). This may assist AHAs to build relationships with other healthcare professions and raise their profile in the broader healthcare team.

Health services also need to address the enduring lack of clarity of AHAs' scope of practice, which, for AHPs, has been a barrier to delegation of patient care (Brown et al., 2020; Mickan et al., 2018). The AHAs in our study shared this view, with the perceived lack of clarity appearing more problematic for multidisciplinary AHAs who work under the delegation of the smaller AHA professions, such as speech pathology and dietetics. This likely reflects the lower levels of experience of AHA delegation of these professions compared with the larger professions, such as physiotherapy and occupational therapy, who have had more time to establish clarity on the roles and responsibilities of their AHA workforce (Lizarondo et al., 2010). This ambiguity will only become more problematic as AHAs' scope of practice expands under the delegation of smaller professions, as observed in speech‐language pathology AHAs in some settings (Frowen et al., 2021; Kiss et al., 2019). Moreover, multidisciplinary AHAs are exposed to multiple sets of team dynamics, discipline‐specific processes, and priorities (Körner et al., 2015). They are therefore in a unique and challenging position that requires negotiation and navigation of these nuanced dynamics and demands, within and across multiple teams. This likely contributes to the lack of clarity around multidisciplinary AHA roles. Health service guidelines informing AHAs' scope of practice and clear competencies, processes, and procedures for delegation to all types of AHAs may improve clarity on AHAs’ roles within and across teams and enhance utilisation of the AHA workforce (Huglin et al., 2021; Pearce & Pagett, 2015).

Education and supervision are required for AHAs to practice to their full potential. Senior AHPS and academics consider the current vocational training inconsistent and insufficient for preparing AHAs to work across all healthcare settings (Mickan et al., 2018). Therefore, health services are required to provide “on‐the‐job” training for AHAs so they can meet the expectations of the workplace (Huglin et al., 2021; Mickan et al., 2018). Responsibility for the provision of training falls to AHPs who are often unprepared and ill equipped to train AHAs (Brown et al., 2020; Moran et al., 2015). As such, it can be difficult for health services to provide consistent and adequate training, and this likely contributes to the inconsistency in educational opportunities reported by AHAs in our study. One possible initiative that may ease this burden on health services is the development of a diploma course that allows AHAs to specialise in certain areas of healthcare (Pearce & Pagett, 2015). Expectations of higher remuneration for AHAs that pursue further education have been noted as a potential barrier to implementing such initiatives (Mickan et al., 2018). Nonetheless, AHAs in our study showed a passion for further learning that advances their skillset, suggesting that there may be demand for these higher‐level educational pathways.

AHAs reported a desire to increase their role in patient care, which is consistent with the needs of the AHP workforce (Somerville et al., 2015, 2018). Although this is encouraging, they also reported feelings of inferiority and frustration when it came to their exclusion from discussions regarding the future of their roles. Some allied health professions and their lead representing organisations are understandably protective of their professional role and may be reluctant to engage AHAs in these discussions due to fear of losing control over decisions regarding AHAs' scope of practice (Huglin et al., 2021; Mickan et al., 2018). However, expanding AHAs' scope of practice is not intended to substitute the role of AHPs, but rather enable AHPs to practice at the higher end of their scope and, in turn, reduce healthcare workforce expenditure (Snowdon et al., 2021; Somerville et al., 2015). This will enhance the capacity of allied health to meet the needs of an ageing population with greater healthcare needs (World Health Organisation, 2015). Moving forward, there needs to be better engagement of the AHA workforce when making decisions about the future of their roles.

Inadequate and inconsistent career progression for AHAs is a long‐standing issue and difficult to address. Currently in the Victorian public health system, there is a three‐level structure for AHAs (Department of Health & Human Services, 2012). The introduction of a fourth level for AHAs seems a logical next step; however, due diligence and genuine engagement with the relevant stakeholder groups, including AHAs, is required to comprehensively plan and implement this potential change (Pearce & Pagett, 2015). Indeed, the implementation of an advanced AHA career structure would only be possible if the previously described recommendations are actioned: increased clarity of the current role and scope of practice of AHAs; appropriate education programmes for AHAs wishing to advance their skills; and meaningful engagement with AHAs to plan and implement AHA workforce enhancement initiatives.

### 4.1 Methodological strengths and limitations

This study involved a broad sample of AHAs from multiple workplace settings, with varying levels of experience, and working with different delegating AHPs. Our focused study aims, approach to data collection, and rigorous team‐based approach to data analysis, meant that we were able to satisfy information power (Malterud et al., 2016) and increase trustworthiness of the results. There are, however, limitations that need to be considered when interpreting the results of our study. First, the AHAs who participated in our study worked in an Australian public health service, which limits the transferability of our results to public healthcare settings with similar characteristics. The experiences of AHAs in the private sector may differ due to different contextual factors and demands. The sample was dominated by hospital‐based AHAs, with less than half working in community settings or mixed settings. The sample was also too small to explore how contextual factors that influence the AHA role varied between AHAs working under the remit of different allied health professions, within uni‐ or multidisciplinary frameworks, or across different settings. However, the aim of this study was to achieve an overall impression of how contextual factors influence the AHA role rather than investigate this for each delegating profession or healthcare setting.

Future research may explore: 1) the transferability of our results to a national and/or global context, 2) how contextual factors vary between AHAs working under the remit of different allied health professions or within different healthcare settings, and 3) how the suggested actions to enhance AHAs' roles can be operationalised, from multiple stakeholders' perspectives, including AHPs, managers, AHAs, and educators.

## 5 CONCLUSION

This study illustrates AHAs' perceptions of the multiple influences and factors that shape their roles. These insights may inform initiatives to better support and optimise AHAs' roles. Some initiatives to promote the utilisation of the AHA workforce to its full scope may include health service‐level identification and promotion of the AHA role; education pathways to support AHAs who aspire to attain advanced skills; meaningful engagement between allied health, policymakers, and AHAs; to make informed decisions about AHAs' future role and scope of practice; and advancement of the AHA career pathway.

## AUTHOR CONTRIBUTIONS

DS conceived the study idea and all authors contributed to the study design. Four authors collected the data (JP, AD, DS, and OK). Three authors contributed to data analysis (OK, DS, and JP). OK and DS drafted the manuscript. All authors provided critical feedback and approved the final version.

## FUNDING INFORMATION

Open access publication of this research was enabled via the agreement between Monash University and Wiley.

## CONFLICT OF INTEREST

The authors declare that there is no conflict of interest.

## Supporting information


Appendix S1

## Data Availability

The data are not publicly available due to privacy or ethical restrictions.
