# Supplementary material for: Allied health assistants' perspectives of their role in healthcare settings: A qualitative study
Source: Health Soc Care Community. 2022 Jun 10;30(6):e4684–93. doi: 10.1111/hsc.13874 (PMC10084421; doi:10.1111/hsc.13874)
Supplement: Supplementary file 1 — Appendix S1 [file HSC-30-e4684-s001.docx]

# Supplementary file 1

## Interview guide

**Please tell me about your role as an AHA.**

*What setting/s do you predominately work in?*

*What types of allied health professionals do you work with?*

*What types of clients do you work with?*

*What types of activities do you do?*

**Please tell me more about the types of activities you spend the greater part/s of your day doing.**

*Activities that are more clinical in nature or require direct contact with patients / carers*

*Activities that do not require direct contact with patients or carers (for examples, administrative tasks, cleaning, maintenance etc.)*

**Please explain how you think these activities do or do not add value to your role as an AHA**

*Which activities do you think are more important for improving patient outcomes or experience and why?*

*Which activities do you think are less valuable and why?*

**Please describe the proportion of your day you think you spend on more important / higher value activities and that you spend on less valuable activities**

*You can describe as a proportion of the day, week or month*

**Please explain what factors you think influence your opportunity to engage in activities that you consider to be higher value**

*Who / what shapes your role?*

*Who refers or delegates high value activities to you?*

*What training or continuing education have you had, or do you need, to support your engagement in high value activities?*

**How do you think systems and processes at your health service could be changed to maximise the value of your role as an AHA?**
